# Cognitive and Physiological Measures in Well-Being Science: Limitations and Lessons

**DOI:** 10.3389/fpsyg.2019.01630

**Published:** 2019-07-12

**Authors:** Benjamin D. Yetton, Julia Revord, Seth Margolis, Sonja Lyubomirsky, Aaron R. Seitz

**Affiliations:** ^1^Cognitive Science Department, University of California, Irvine, Irvine, CA, United States; ^2^Cognitive Science Department, University of California, Riverside, Riverside, CA, United States

**Keywords:** positive affect, negative affect, well-being, interventions, prosocial behavior, cognition, EEG, physiology

## Abstract

Social and personality psychology have been criticized for overreliance on potentially biased self-report variables. In well-being science, researchers have called for more “objective” physiological and cognitive measures to evaluate the efficacy of well-being-increasing interventions. This may now be possible with the recent rise of cost-effective, commercially available wireless physiological recording devices and smartphone-based cognitive testing. We sought to determine whether cognitive and physiological measures, coupled with machine learning methods, could quantify the effects of positive interventions. The current 2-part study used a college sample (*N* = 245) to contrast the cognitive (memory, attention, construal) and physiological (autonomic, electroencephalogram) effects of engaging in one of two randomly assigned writing activities (i.e., prosocial or “antisocial”). In the prosocial condition, participants described an interaction when they acted in a kind way, then described an interaction when they received kindness. In the “antisocial” condition, participants wrote instead about an interaction when they acted in an *un*kind way and received *un*kindness, respectively. Our study replicated previous research on the beneficial effects of recalling prosocial experiences as assessed by self-report. However, we did not detect an effect of the positive or negative activity intervention on either cognitive or physiological measures. More research is needed to investigate under what conditions cognitive and physiological measures may be applicable, but our findings lead us to conclude that they should not be unilaterally favored over the traditional self-report approach.

## Introduction

Most people report wanting to be happy (Diener, 2000) – that is, to feel satisfied with their lives and to experience frequent positive emotions and infrequent negative emotions (Diener et al., 1999). A growing number of studies show that happiness correlates with, predicts, and causes many positive outcomes, including success in work (e.g., performance and salary), social relationships (e.g., number of friends and social support), and health and coping [e.g., physical symptoms and longevity (Prochaska et al., 2012)]; for reviews, see (Lyubomirsky et al., 2005; Diener et al., 2017; Walsh et al., 2018). Happy people also tend to show more positive perceptions of themselves and others, greater sociability and likability, more prosocial behavior, and superior creativity (Lyubomirsky et al., 2005).

According to Google Trends (Google Trends, 2019), searches for well-being have increased more than 500% since collection began in 2004. Given the clear benefits of happiness, it is unsurprising to see rising interest in personal well-being and methods to increase it. Literature in the growing area of well-being science points toward positive activity interventions (PAIs), such as writing letters of gratitude (e.g., Emmons and McCullough, 2003; Lyubomirsky et al., 2011), and practicing kindness (e.g., Dunn et al., 2008; Chancellor et al., 2018), as simple behavioral strategies to promote well-being, many of which have been empirically validated (Sin and Lyubomirsky, 2009; Bolier et al., 2013; Layous and Lyubomirsky, 2014). These PAIs have the potential to improve affect, and in turn, promote positive health and well-being outcomes without the use of drugs, costly or stigmatizing treatment, or significant behavioral changes. However, for such interventions to become useful and trusted tools for clinical or public use, the ability to detect their efficacy is critical.

The current state of the art measurement of happiness, and of PAI efficacy, is through self-report. However, self-report variables, even those with decent reliability and validity, are notoriously biased (Schwarz, 1999; Dunning et al., 2004), especially toward socially desirable responses (Velicer et al., 1992; Mezulis et al., 2004; Van De Mortel, 2008) – for example, toward appearing to be happier. Further, individual differences such as sex and culture serve as moderators or create additional variance (Lindsay and Widiger, 1995; Beaten et al., 2000). This has led researchers to search for “objective” measures of happiness that do not rely on self-report, like Facebook status updates (Chen et al., 2017) or Duchenne smiles (Harker and Keltner, 2001). Measures of physiology like electroencephalography (EEG) or heart rate monitoring, as well as cognitive tasks that tap into domains like memory, attention, and perception, are considered less biased by the social influences that plague self-report. Although these measures can be noisy, they are considered (by some) as more objective measures of underlying emotional and cognitive processes (Calvo and Mello, 2015).

Here we aimed to leverage recent advancements in low-cost, readily available physiological measurement devices and a large body of cognitive psychology research to determine the practical utility of cognitive and physiological measures in assessing the effects of a positive versus negative activity intervention. We aimed to verify whether these measures were indeed simple to implement, robust, and predictive for well-being research, thereby providing alternative measures to self-reported well-being levels.

In the current study, students were administered either a PAIs (writing about gratitude and recalling a kind act) or a negative activity intervention (writing about ingratitude and recalling an unkind act). Before and after the intervention, the participants completed both standard psychological measures of their current affective states, as well as several tasks designed to quantify the cognitive domains of memory, attention, and high-level perception. Additionally, physiological measurements of the central (EEG) and autonomic (heart rate, skin temperature, galvanic skin conductance) systems were recorded. This work builds upon our previous findings (Revord et al., 2019), which reported the self-report psychological outcomes of the same positive and negative activity interventions.

### Cognitive Outcomes of Positive Activities

Although the well-being outcomes of positive activities like gratitude and kindness are widely reported (see Layous and Lyubomirsky, 2014, for a review), the cognitive outcomes of positive activities have been largely unexplored. Here we focus on the cognitive domains of attention, memory, and situation construal (i.e., high-level perception).

Numerous studies have investigated how attention may be biased toward emotionally salient information that matches one’s current emotional state. For example, in one study, induced negative affect generated an attentional bias that favored sad faces (Gotlib et al., 2004). Similarly, individuals high in trait anxiety have been found to pay more attention to threat (Derryberry and Reed, 1998; Fox et al., 2001). However, these results are muddied by differing effects across age groups (Mather and Carstensen, 2003), and unreliable tasks, such as the dot probe (Neshat-Doost et al., 2000; Schmukle, 2005). Positive activities such as practicing gratitude or kindness may involve deeper, more meaningful, or more complex (e.g., self-reflective or other-focused) emotions, and hence other outcomes than those induced by simple positive affect inductions may be expected. Furthermore, attention is multi-faceted, with multiple components (Mirsky et al., 1991), and support for modulation of other attentional processes with positive interventions is scarce. For example, one study reported no effect of happiness or sadness inductions on alerting, orienting, or executive attention, but found that participants induced to feel sadness exhibited reduced intrinsic alertness (Finucane et al., 2010).

Additionally, it is unclear what other aspects of cognition, such as perception and memory, may be impacted by PAIs. Certainly, trait levels of some positive constructs are related to cognitive biases. For example, those with higher subjective happiness appear to show a self-enhancing bias (J. Y. Lee, 2007). Happy people are also more likely to report more frequent and intense daily happy experiences (Otake et al., 2006), to recall more positive life events and fewer negative life events (Seidlitz and Diener, 1993), and to report more intense and enduring reactions to positive events (Seidlitz et al., 1997). Even when they do not report more positive events, happy people tend to think about negative events in more adaptive terms and to describe new situations in more positive ways (Lyubomirsky and Tucker, 1998). Individuals with higher levels of life satisfaction show superior ability to accurately retain and update positive memory (Pe et al., 2013). Other studies have revealed people high in trait gratitude to have a positive memory bias (Watkins et al., 2004), and those high in trait optimism to show a greater attentional bias for positive than negative stimuli (Segerstrom, 2001). On the opposite side of the spectrum, people who are depressed tend to exhibit a memory bias that favors negative words (Denny and Hunt, 1992).

Although trait-level affect appears to impact cognition, some experimental work also links positive activities with changes in cognition. For example, a 3-day loving-kindness meditation training impacted how easily participants associated positivity with neutral stimuli (Hunsinger et al., 2013). Furthermore, the find-remind-and-bind theory postulates that gratitude shifts cognitive perspective – including situation appraisals, short-term changes in cognition, motivations, and behaviors (Algoe, 2012).

We hypothesized that engaging in a prosocial writing activity, relative to an antisocial writing activity, would drive cognitive biases in several areas: (1) leading people to attend more to positive stimuli and less to negative stimuli; (2) prompting people to have better memory for positive stimuli and worse memory for negative stimuli; and (3) leading people to construe a variety of positive, negative, and neutral situations more positively.

### Physiological Outcomes of Positive Activities

Detecting brain and body changes due to PAIs would further validate their efficacy. Presumably objective measures, such as physiological recording, provide a less biased alternative to self-report emotional state measures, which can reflect personal beliefs or correlate due to shared method variance. Here we aimed to use low cost, commercially available, wireless devices to detect changes in the central nervous system (electroencephalogram) and autonomic nervous system (ANS) (skin conductance, skin temperature, blood volume pulse). These devices are readily feasible to use outside of the lab environment, and thereby potentially scalable in field settings, such as clinical, organizational, and educational contexts.

Electroencephalography is a physiological technique in which voltage measuring electrodes are placed in various locations on the scalp surface. While this technique has high temporal resolution, spatial resolution is low, and signals represent the combined activation of millions of cortical neurons in a broad area under each electrode, as filtered through the thick scalp bone. Although much research focuses on inaccessible sub-cortical areas in the generation, expression, and regulation of emotion (see Phelps and LeDoux, 2005, for review), there is evidence linking prefrontal cortical regions, and their subsequent EEG patterns, to emotional processing (Davidson, 2004). Multiple studies report that alpha power asymmetries between frontal electrodes are correlated with self-report variables of affect, such that greater left activation is linked to positive affect, and greater right activation is linked to more negative affect (Tomarken et al., 1992; Wheeler et al., 2007). Emotions induced by positive (joy) versus negative (sad) music are also detectable from these frontal electrodes (Schmidt and Trainor, 2001; Naji et al., 2014). Similar to the interventions used in the current study, Hinrichs and Machleidt (1992) asked participants to recall positive and negative life events, while recording EEG activity. In contrast to the aforementioned studies, they found less asymmetry in alpha activity across hemispheres at frontal, temporal, and occipital sites during the recall of joyful events, and more alpha asymmetry at the same electrode positions during recall of sad and anxiety-provoking events.

Although findings regarding the effects of emotion on EEG lateralization are mixed, consensus in the field is that lateralization in frontal sites is related to the personal experience of emotion (rather that the perception of others’ emotion) and that perception, regardless of valence, may be visible in posterior electrode sites (Davidson, 1993). In this paper, we investigate the efficacy of using EEG asymmetries in gauging affective state changes in response to positive and negative activity interventions, as well as taking a more exploratory, data-driven, machine learning approach to identify possible new EEG correlates of the effects of such interventions.

The ANS regulates body functions not under conscious control, such as respiratory, cardiac, exocrine and endocrine activity. The ANS is in continuous balance between a more parasympathetic (fight or flight) or sympathetic (rest and digest) state. The profile of ANS activity is influenced by emotion; positive affect is characterized by increased parasympathetic nervous system activity, and negative affect results in parasympathetic withdrawal and sympathetic activity (McCraty et al., 1995). The state of the ANS may be indexed by various physiological variables, such as heart rate, heart rate variability (extracted from electrocardiogram or blood pressure volume measures), skin temperature, skin conductance, and respiration rate/depth.

Considerable debate over the years has focused on the effect of emotions on the ANS. Some theories, such as that of Schachter and Singer (1962), state that only the arousal level (i.e., intensity) of an emotion can be detected in the periphery, while others theorize both emotional valence (happy/sad) and arousal induce detectable ANS changes (Lazarus, 1991). Here, we pose a similar question: Does a positive versus negative activity intervention lead to ANS changes as measured by skin conductance, skin temperature, and blood pressure volume?

Mounting evidence suggests this is the case: In pioneering work by Ekman et al. (2013), people were asked to recall autobiographical events that elicited one of six emotions (surprise, disgust, sadness, anger, fear and happiness) while recording physiology. Significant differences in skin temperature, heart rate and skin conductance were observed among these emotions. Similarly, Rainville et al. (2006) asked participants to vividly recall emotional events while collecting cardiac and respiratory activity. They found emotions of happiness, sadness, fear, and anger all drove separate patterns of autonomic activity; a univariate analytic approach revealed that anger-fear and fear-sadness were the most discriminable discrete emotions. Kop et al. (2011) noted increased low frequency heart rate variability (LF-HRV) but not high frequency (HF-HRV) in response to recall of happy memories, as well as increased alpha power in right frontal EEG. A correlation was also observed between ANS (HF/LF HRV ratio) and CNS (EEG right frontal alpha). Haiblum-Itskovitch et al. (2018) used an art-making intervention to induce positive emotion, and found decreases in parasympathetic arousal, as indexed by HF-HRV.

Across studies, different variables (HF-HRV, LF-HRV, LF/HF Ratio, skin temperature, skin conductance) have been found to track emotion changes, suggesting a complex multivariate and non-linear relation between emotion and cognition and the need for more advanced analytic techniques. Advancements in affective computing, which fuses multimodal physiology signals using a data-driven machine learning approach (Jerritta et al., 2011; D’mello and Kory, 2015), have helped bridge this gap. For example, Kim et al. (2004) used heart rate measures, skin temperature, and skin conductance to classify three emotions with an accuracy of 78% and Wagner et al. (2005) classified four emotions at 92% accuracy using features derived from dimensional reduction of skin conductance, electromyogram, respiration, and blood pressure volume. Many methods also include EEG features as predictors, with accuracies ranging from 55 to 82% (Koelstra et al., 2010; Verma and Tiwary, 2014; Yin et al., 2017) for emotions induced by music, and 70% for recalled emotional events (Chanel et al., 2009). Work from this field stresses the importance of large sample sizes, filtering artifacts, and comparing an emotional state to a within subjects baseline, as variance across participants can be large, and ceiling and floor effects are expected in many physiological parameters (Calvo and Mello, 2015).

In our second experiment, we aimed to investigate the effects of a positive and negative intervention on the brain and body though physiological measures of electroencephalogram (EEG), galvanic skin conductance (GSC), skin temperature (ST), and blood volume pulse (BVP) – containing heart rate variability (HRV) information. We explicitly test two hypotheses motivated by previous research and theory – namely, expecting (1) greater frontal EEG asymmetry in the positive condition and (2) lower HF-HRV and higher LF-HRV in the positive condition. We also present analyses that follow a more exploratory approach, where machine learning is used to elucidate the effect of the two interventions on central and ANS measures.

## Materials and Methods

### Participants

Two-hundred forty-five undergraduates (Experiment 1: *N* = 159, *M*_age_ = 19.66; *SD* = 3.08; Experiment 2: *N* = 86, *M*_age_ = 19.32, *SD* = 2.02) were recruited from a participant pool across introductory psychology courses at the University of California, Riverside. Students were invited to participate via an online portal, and were reimbursed in course credit for their time. Written student consent was obtained in accordance with the University of California, Riverside Institutional Review Board. Inclusion criteria required that all participants be over age 18 and fluent in English, with no history of mental illness and currently not taking any medication. Pooling across all participants, 64.8% were female, and 91.1% were right-handed. They reported their ethnicity as 1.6% American Indian/Alaskan Native, 17.9% Asian, 15.2% Black/African American, 0.8% Hawaiian/Pacific Islander, 7% White, 34% Hispanic/Latino, 17% more than one, and 5% other. None of the reported demographics significantly related to intervention condition.

### Procedure

Experiment 1 involved a single in-lab session in which participants filled out an online baseline questionnaire, including a consent form, demographic information, and psychological measures. They then completed baseline cognitive tasks, including word memory recall, word memory recognition, situation construal, and a “word find” with positive, negative, and neutral words. After completing the pre-intervention baseline measures, participants were randomly assigned to one of two intervention conditions: a positive writing condition (i.e., the prosocial writing activity; *n =* 78) or a negative writing condition (i.e., the antisocial writing activity; *n* = 81). Each writing condition involved a free response to a recalled gratitude (vs. ingratitude) and recalled prosocial (vs. “antisocial”) prompt of the same valence. Exact wording of each prompt appears in Supplementary Material (S1 Text). After each prompt was shown, students were unable to advance to the next screen until at least 5 min had passed and were limited to 10 min total. All subjects were able to complete the writing prompt within the 10-min limit. Furthermore, they were instructed to describe a target act (e.g., a kind or unkind act someone did to them) using specific language, discuss how it is still affecting them, and report how often they think about the act. Participants were also instructed to use any format, and not worry about perfect grammar and spelling. At post-intervention, they completed the same psychological and cognitive variables (in the same order) as at baseline and then were debriefed. Questions and tasks proceeded in the same order for all participants. The total time for the experimental session (after initial participant setup) was approximately 1 h, with 40 min between pre-and post-test.

Experiment 2 involved one online and one in lab session over an 8-day period. On Day 1, students completed a demographics and trait affect questionnaire (not analyzed). On Day 8, they came into the lab and were hooked up to EEG recording equipment and the Feel^*TM*^ wristband (which records galvanic skin conductance [GSR], skin temperature [ST] and blood volume pulse [BVP]). The rest of the lab session proceeded as in Experiment 1. To control for possible order effects, in Experiment 2, the order of tasks/questionnaires (and items within questionnaires) were counterbalanced within condition using a Williams design to eliminate first-order effects.

### Materials

All questionnaires, cognitive tasks, and writing prompts for the in-lab session were presented using Psychtoolbox (Kleiner et al., 2007) for Matlab^TM^ (The MathWorks Inc., 2015). All online questionnaires were presented via Qualtrics, 2019.

#### Positive and Negative Activity Intervention

Students were randomly assigned to either a positive (prosocial) writing condition or a negative (“antisocial”) writing condition. The prosocial writing condition was designed to elicit a strong positive emotional response, and included both recalling something kind that someone else had done and recalling something kind that the participants themselves had done (cf. Layous and Lyubomirsky, 2014). The antisocial writing condition was designed to complement the prosocial condition, and included the opposite prompts – namely, recalling something unkind that someone else had done and recalling something unkind that the participants themselves had done.

#### Self-Report Variables

Validated questionnaires addressed multiple psychological states, which included measures of positive and negative affect (Affect-Adjective Scale; Emmons and Diener, 1985), elevation (Haidt, 2003), gratitude (Gratitude Adjective Checklist; McCullough et al., 2002), negative social emotions (i.e., guilt, indebtedness), optimism (Life Orientation Test-Revised; Scheier and Carver, 1985), state self-esteem (State Self-Esteem; (Rosenberg, 1965), meaning in life (Meaning in Life Questionnaire; Steger et al., 2006), and psychological needs (i.e., connectedness, autonomy, and competence; Balanced Measure of Psychological Needs: Sheldon and Hilpert, 2012). Questionnaires were chosen based on their successful use in previous research (Sheldon and Lyubomirsky, 2006; Nelson et al., 2015, 2016; Layous et al., 2017) in order to track changes in constructs that were expected to shift after positive and negative writing tasks. Another candidate questionnaire, the Gallup-Healthways Well-Being Index, was omitted due to its focus on the more trait like aspects of wellbeing that were not expected to change across the relatively short experimental timeline. Each questionnaire was administered before and after the writing intervention. Full results of these self-report variables are presented elsewhere (Revord et al., 2019), but are compared to cognitive and physiological measures here.

#### Cognitive Variables

##### Word memory

To assess memory for words of different valence, both before and after the writing intervention tasks, participants were serially presented with 16 positive words (e.g., cheer, merry, beautiful), 16 negative words (e.g., deserter, poverty, devil), and 16 neutral words (e.g., statue, inhabitant, scissors) and instructed to remember them (passive memorization, with no response requested). A different set of words was used pre- and post-intervention. This segment of the task lasted approximately 1.5 min, where words were presented for 1 s with a 2.75–3.25 s jittered inter-stimulus-interval (to facilitate the physiological analyses). Words were selected from the Affective Norms for English Words data set (ANEW; Bradley and Lang, 1999), such that positive words had an average valence of 7.25, negative words had an average valence of 2.75, and neutral words had an average valence of 5.00. Arousal ranged from 5.08 to 5.12 across valence conditions.

Ten minutes after word presentation, recognition memory was tested by presenting the 48 original words in random order, as well as 48 distractor words (16 positive, 16 negative, 16 neutral) in a 8 × 12 grid. Students were asked to click on all the words on the screen that they recognized. They could correctly click on old words (a “hit”), correctly choose not to click on new words (a “correct rejection”), mistakenly click on new words (a “false alarm”), or fail to click on old words (a “miss”). Average completion time for this segment of the task 5 min. *D-prime* and *C*, commons measure of sensitivity stemming from signal detection theory (see Nevin, 1969, for review) was used to quantify recognition memory and bias for each word valence separately. Briefly, *d-prime* provides an estimate of the distance between the distribution of the signal (correct rejections and hits) and the distribution of the noise (misses and false alarms), and *C* measures how biased a participant is to respond old versus new on average. For calculation purposes, when the hit or false alarm rate was equal to zero, d-prime was set to 1/2*N* and when the hit or false alarm rate was equal to 1, d-prime was set to 1−1/2*N*. Larger scores on *d’* indicate greater accuracy.

##### Situation construal

In the situation construal task, participants were presented with 6 photos – two positive situations (e.g., a couple kissing), two negative situations (e.g., a person wading through a polluted ditch), and two neutral or ambiguous situations (e.g., bikers jumping train tracks at high speed, with a train approaching in the background). Images were selected from the International Affective Picture System (IAPS; Lang, 2005). On the IAPS valence ratings (1 = *most negative*, 9 = *most positive*), the negative images had an average valence of 2.5, the ambiguous images had an average valence of 5.0, and the positive images had an average valence of 7.5. A different set of 6 images were presented at each test (stimuli randomly assigned to either test). Positive images consisted of slides 2352, 4597, 8090, and 8300; negative images consisted of slides 9342, 9530, 9042, and 6561; and ambiguous images consisted of slides 8475, 2595, 2749, and 8160. The time to complete task was approximately 4 min.

Participants were asked to rate each of these situations using 10 items from the Riverside Situational Q-sort (Funder et al., 2000; Funder, 2016) (1 = *not at all*, 7 = *extremely*). They included 5 positive items (i.e., “situation is playful,” “situation is potentially enjoyable,” “situation might evoke warmth or compassion,” “situation is humorous or potentially humorous (if one finds that sort of thing funny),” and “situation might evoke warmth or compassion”), and five negative items (i.e., “situation contains physical threats,” “someone is being abused or victimized,” “someone is unhappy or suffering,” “situation would make some people tense and upset,” and “someone is under threat”). No time limit was imposed, and images were displayed on screen until all 10 questions were complete. Across measurements, Cronbach’s α ranged from 0.82 to 0.85 for the positive items and from 0.85 to 0.86 for the negative items. Two measure for each image valence were extracted:

##### Response normalized

The response of each item (question) was normalized from the range (1–7), to (–1, +1). Negative items were flipped such that all values above 0 represented positive appraisal of the image. Within each image valence, we took the average of all normalized responses. This gave us a measure of how positively each participant construed each image valence, where 1 is more positive, and –1 is more negative. If the positive intervention increases positive construal, we expect an increase in this measure across all image valences.

##### Reaction time

Alternatively, the intervention may increase or decrease how fast participant respond to each item. Therefore, the reaction time, averaged within each image valence is also reported.

##### Word find

To assess the degree to which participants attended to positive versus negative content (Mirsky et al., 1991; Raz and Buhle, 2006), they completed a “word find” task. On a sheet of paper, all participants were presented with the same 15 × 15 word find. The nine words in the word search comprised three positive (e.g., sweetheart), three negative (e.g., slave), and three neutral (e.g., fabric) words, each displayed in pseudorandom positions on screen. A different set of words was used pre- and post-intervention. When a word was found, participants were instructed to circle the word, then click the word on the screen. This procedure allowed us to randomize the order in which participants saw the to-be-found words and eliminate effects of systematic searching in the order of word presentation. Subjects completed the task in approximately 6 min.

##### Average order

The first primary outcome of the word find was the average order of finding each word valence type. For example, out of the nine words, the first word found is 1, last word found is 9 and the average will be 5. By taking the average of the order in each valence group, we obtained an estimate for the time it took to find that valence group, with a number below 5 signifying faster than average, and above 5 signifying slower than average.

##### Average time found

The second outcome of the word find was the average time taken from beginning the task to find the words in a valence group. Lower average time found indicates that a valence group was found more quickly, as measured in milliseconds.

#### Physiological Measures

##### ANS variables

To measure autonomic activity, we used the Feel^*TM*^ wristband, a to-be-released commercial “emotion tracker” (Sentio, Athens, Greece). The device contains skin conductance, skin temperature, and blood volume pulse sensors, as well as a 3-axis accelerometer/gyroscope, all sampled at 500 Hz. Signals were denoised for movement, sweat, and other artifacts using Sentio’s proprietary algorithms. Along with the denoised signals, the wristband also gave the interpolated inter-beat interval, as extracted from the BPV sensor (4 Hz sample rate), and the tonic/phasic decomposition of the skin conductance signal (20 Hz). The wristband’s internal clock was synchronized to the clock of the stimulus presentation computer, thereby allowing for event driven analysis. Raw signals were transformed into 5 “feature” signals (see Table 1 for details).

**TABLE 1:** Features extracted from the Feel^*TM*^ wristband.

**Feature**	**Base Sensor**	**Transformation**
High Frequency HRV	Blood Volume Pulse	Cubic Spline Interpolation of RR signal, band power 0.15–0.4 Hz
Low Frequency HRV	Blood Volume Pulse	Cubic Spline Interpolation of RR signal, band power 0.04–0.15 Hz
Tonic GSR	Galvanic Skin Conductance Sensor	Sentio Proprietary Algorithm
Phasic GSR	Galvanic Skin Conductance Sensor	Sentio Proprietary Algorithm
Skin Temperature	Temperature Sensor	Identity

Physiological recording took place throughout the experiment, with many possible events on which to base physiological analysis. Following Calvo and Mello (2015) and Calvo et al. (2016) emotion classification paradigm, we chose events with low motion artifacts (i.e., not during writing or during the word find). As a result, the analyzed events were (1) emotional memory word presentation, (2) situation construal question presentation, and (3) presentation of each question during the self-report psychological measures. For periods when the stimulus was present on screen, the amplitude of the signal was extracted for each feature, as well as peak-to-peak. We then tested whether the pattern of changes in physiology across the intervention (as measured at specific event times) differed by intervention type.

##### Electroencephalogram

A 14-channel wireless EEG device (Emotiv Epoch; Emotiv, 2019) was modified to include an infracerebral electrode cap, better quality Ag/AgCl electrodes, a more comfortable form factor, and alternate electrode placement locations corresponding to the 10–20 placement system. Modifications were completed in lab using the an upgrade kit and directions from EASYCAP (Debener et al., 2012; Easycap GmbH, n.d.). In initial piloting, we noticed the EEG device was dropping samples, sometimes at random, and therefore syncing the signal to stimulus presentation was impossible. To rectify this, we wired a photo-diode into two channels of the EEG device (“sync channels”) and placed this photo diode on the stimulus presentation computer. For each stimulus presentation, a small dark square was flashed under the diode, which registered as a sharp rising or falling edge on these sync channels. The 12 leftover channels were placed at F4, F7, Fz, F5, F8, C3, Cz, P7, P3, Pz, P4, P8, Oz (with reference sensors common mode sense and driven right leg at Afz and Fcz). Sampling rate was 128 Hz.

Electroencephalography signals were band passed filtered between 0.1 and 60 Hz. The rising and falling edge of the sync channels were detected and assigned stimulus event markers and then these channels were removed. Channel data were visually inspected and any bad channels were removed; channels were then re-referenced to the average of all good channels. Noise from movement, sweat, and other sources were of concern, especially with the relatively low signal to noise ratio of this device. To remove eyeblink artifacts, we transformed data using Independent Spectral Analysis (ICA), and if a channel with clear eyeblink artifact pattern was observed, it was removed. Data were then transformed back to the original domain.

To remove other artifact segments from a channel (e.g., sweat, movement, electrode disconnection, electromagnetic interference), filtered versions of each channel were created in the delta, alpha, theta, alpha and beta bands 1–4 10, 4–7, 8–15, and 16–31 Hz band filters (filter channels). The envelope of each of these channels was also extracted using a Hilbert transform (filter envelope channels). Segments of each channel separately were marked as artifacts if any of the following conditions were true in a 0.3 s sliding window: (1) absolute value of original channel above 400 μV, (2) peak to peak amplitude greater than 400 μV, or (3) amplitude of filter envelope channels greater than 40 μV. These filter parameters were chosen so to minimize the inclusion of artifacts while maximizing the retained good signal, as determined by visual inspection.

From each channel, and for each of the “stimulus on” events of the situation construal task, emotional memory task, and self-report variables (as for the ANS measures), we extracted a number of features (Table 2). The Python toolbox pyEEG (Bao et al., 2011) was used to extract some of the more complex features. These features where averaged for each participant and channel group, where channel groups were defined as F = [F7, F8, Fz], C = [Cz, C3, C4], P = [P3, P4, P7, P8, Pz], O = [Oz]. However, given the number of variables, and the low sample size, overfitting was a concern. Principle component analysis (PCA) was employed to reduce the number variables from 24 to 5 components (per task, per subject). Directly applying PCA to the non-grouped channels produced the same results. Asynchronous measures, due to their prior theoretical predictions, were not reduced by PCA, and analyzed separately.

**TABLE 2:** EEG features extracted from EEG windows.

**Feature**	**Variants/Channels**	**Transform from EEG**
Band Power (RMS)	For all channels: Theta (4–7 Hz), Alpha (8–15 Hz), Beta (16–31)	For each band: band pass filter + Root of mean squared signal (RMS).
Hurst Exponent	All Channels	As in pyEEG
Petrosian Fractal Dimension	All Channels	As in pyEEG
Hjorth Fractal Dimension	All Channels	As in pyEEG
Alpha Asynchrony	F3–F4, F7–F8, P7–P8	Difference in alpha RMS power between electrodes on left and right side.

### Analysis

We took a multi-faceted approach to our analyses, employing both standard regression models and several machine learning techniques. All cognitive variables were initially analyzed using univariate regressions (with measures first *z*-score normalized), where the difference between pre-and post-intervention value (“Difference”) of each univariate cognition measure is predicted by the intervention condition (“Intervention,” dummy coded: positive = 1) and an intercept. A significant positive intercept (for positively coded measures) provides evidence for an overall increase in the cognitive measure across interventions, which may be attributable to test-retest effects, and is not of primary interest. A significant negative intercept indicates a reduction in that measure in the positive intervention condition. A significant “Intervention” coefficient represents a significant difference in pre-post change between the negative and positive intervention conditions, and evidence for an effect of the intervention on physiology. Familywise error rate adjustment is applied (Bonferroni) for each variable group (cognitive, Autonomic, EEG).

We built on the standard analysis with a number of machine learning techniques. Generally, the goal of machine learning is to obtain a non-linear mapping between some set of inputs X and a set of outputs Y. This framework has the advantage over simpler modeling techniques (such as ordinary least squares regression) in that many variables can be included in the predictive set X, and techniques such as cross validation and regularization will automatically select the most relevant variables and their interactions, to predict Y, while controlling for overfitting.

If cognitive or physiological variables are differentially affected by the intervention conditions, then they will show opposing patterns of change across the intervention. We used change in the psychological variables to classify whether a participant was in the positive or negative intervention condition. If accuracy is above chance, then it can be concluded that the intervention paradigm produced a physiological response. Further, in simpler models like logistic regression, the predictivity of each variable is reflected in its coefficient.

We ran four types of machine learning models: logistic regression, support vector machines (SVM; Corinna and Vladimir, 1995), adaptive boosted SVM (Freund and Schapire, 1999), and random forests (Liaw and Wiener, 2002). L1 regularization (Ng, 2004) was applied to all models to limit overfitting. Additionally, for logistic regression, regularization has the effect of driving non-predictive variable coefficient to zero. If a coefficient is driven to zero, it does not help predict intervention type, and suggests that this variable is unaffected by the intervention type.

A cross-validation scheme was used to limit overfitting and pick the optimal amount of regularization. The procedure is outlined below:

1. The data were split into *training* (80%) and *test* (20%) sets.
2. The *training* set was then split into thirds.
3. On two-thirds of the data we trained a set of models with different regularization values (e.g., 10^–3^, 10^–2^, 10^–1^, 10^0^, 0, 10^1^), and validated on the remaining third.
4. The one-third splits were then shuffled so that each split was used for both training and validation.
5. The regularization that lead to the best validation accuracy in steps 3 and 4 was selected, and a model was then fit to the entire *training*, and tested on the *test* set.
6. The entire process (1–5) was repeated 20 times to ensure that different *training/test* splits did not bias results.

We report the mean and standard error of training accuracy and test accuracy (from step 5), across the 20 runs. For logistic regression, parameters of the best fitting model among the 20 runs are reported, although it should be noted that parameter value changes were minimal with each run.

An alternative method to validate the physiological measures is to consider the concurrent validity between physio and self-report measures across our intervention. We therefore ran univariate ordinary least squares regression models with each of the 14 self-report measures as the DV, and IVs as all variables from (a) cognitive measures (both studies combined) and (b) physiological variables during each task (i.e., separate models for the set of each variables from Situation Construal, Questionnaire, Emotional Memory). The fit of the model was quantified (F statistic) and only *p*-values less than the Bonferroni corrected alpha level of 0.0036 were considered. Note, however, that well-being is a complex construct, and a simple relation between a set of physio/cognitive measures and a single self-report measure gives an incomplete picture of physiological/cognitive efficacy. Thus, we gave more weight to the machine learning approach.

## Results

### Univariate Regression Analysis

#### Psychological Self Report Variables

**FIGURE 1:**
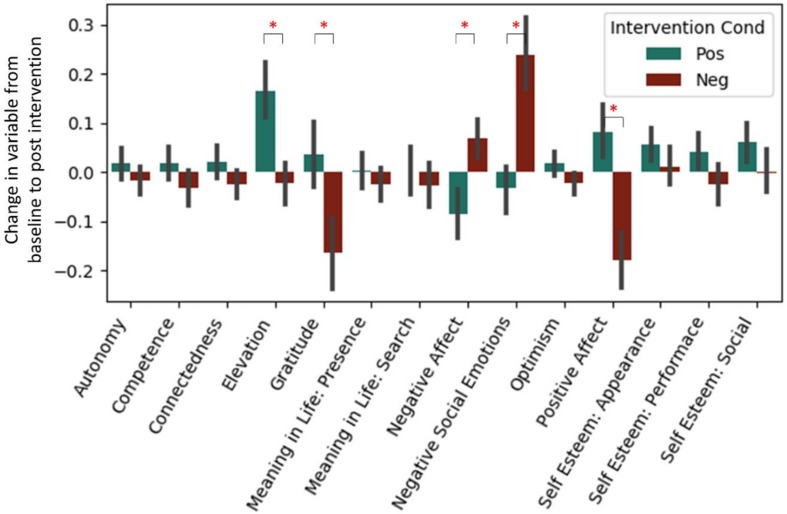
Standardized change between pre and post-intervention for each self-report variable. Each intervention type shown separately. Data from Experiment 1 and 2. ^*^Represent significant difference in pre→post-change between intervention types after Bonferroni correction.

Analyses of our psychological self-report variables appears in a previous article (Revord et al., 2019), but are also briefly summarized here (see Figure 1). A regression predicting change in each measure from baseline to post-test from the intervention type was run for both studies combined (Table 3). After a Bonferroni correction for the 14 measures considered (α = 0.0036), we found in the positive intervention condition significant increases in elevation and self-esteem (appearance and social subscale), as well as a significant decrease in negative affect. Significant decreases in gratitude and positive affect, and a significant increase in negative social emotions, were observed in the negative condition. The positive and negative intervention conditions significantly differed in pre-post change for elevation, gratitude, negative affect, negative social emotions, and positive affect variables.

**TABLE 3:** Standardized mean change from pre-test to post-test for self-report variables.

**DV**	**Change Across Negative Intervention**		**Change Across Positive Intervention**		**Difference between intervention conditions**	
			
	**Mean change**	***p***	**Mean change**	***p***	***p***	**Mean change**
Autonomy	–0.016	0.308	0.018	0.245	0.034	0.123
Competence	–0.033	0.059	0.019	0.285	0.052	0.037
Connectedness	–0.024	0.131	0.021	0.188	0.045	0.046
Elevation	–0.022	0.401	0.166	< 0.001^*^	0.188	< 0.001^*^
Gratitude	–0.162	< 0.001^*^	0.037	0.298	0.199	< 0.001^*^
Meaning in Life: Presence	–0.025	0.152	0.003	0.852	0.029	0.252
Meaning in Life: Search	–0.027	0.258	0.001	0.952	0.028	0.401
Negative Affect	0.068	0.004	–0.085	< 0.001^*^	–0.153	< 0.001^*^
Negative Social Emotions	0.24	< 0.001^*^	–0.033	0.303	–0.273	< 0.001^*^
Optimism	–0.023	0.049	0.018	0.127	0.041	0.014
Positive Affect	–0.179	< 0.001^*^	0.081	0.004	0.26	< 0.001^*^
Self Esteem: Appearance	0.011	0.547	0.056	0.002^*^	0.046	0.068
Self Esteem: Performance	–0.025	0.179	0.042	0.028	0.067	0.012
Self Esteem: Social	–0.001	0.976	0.062	0.003^*^	0.062	0.031

The regression model was run twice with difference dummy code for intervention type. Change Across Negative Intervention represents the intercept when the positive intervention is coded as Pos = 1 and represents mean change in each DV across the negative intervention (when variables are standardized). Change Across Positive Intervention is the intercept when Pos = 0, and the Difference Between Intervention Conditions is the standardized regression coefficient that represents the effect of intervention condition (i.e., the difference between negative and positive conditions). ^*^Represents significance after Bonferroni correction. DV = dependent variable.

#### Cognitive Variables

**FIGURE 2:**
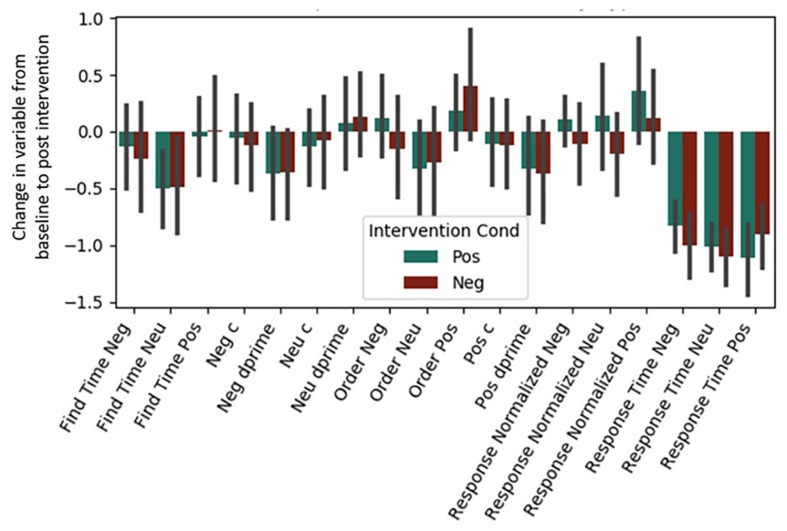
Post-intervention changes in cognitive variables by intervention type. Neu = Neutral. Pos = Positive. Neg = Negative. No significant differences were found across intervention condition. C and d-prime are measures from signal detection theory (see Section “Materials and Methods”).

Both Experiment 1 and 2 included cognitive variables, and the regressions presented below were fit to the combined dataset (see Table 4 and Figure 2). Regressions for our two individual datasets are presented in Supplementary Material (see Supplementary Figures S1, S2 and Supplementary Tables S1, S2). Bonferroni corrections were made for the 18 measures considered (α = 0.0028). Due to test-retest effects, increases in the general speed and accuracy of responses across both conditions were expected. We observed these effects for (1) positive words’ d-prime, (2) response time for the situation construal task, and (3) find times for negative words in the word find (all *p* < 0.0028). A significant intervention coefficient provides compelling evidence that the intervention produced differences in cognitive outcomes; however, no significant intervention effects were found.

**TABLE 4:** Standardized mean change from pre-test to post-test for cognitive tasks.

**Task**	**DV**	**Change Across Negative Intervention**		**Change Across Positive Intervention**		**Difference between intervention conditions**	
				
		**Mean change**	***p***	**Mean change**	***p***	**Mean change**	***p***
Situation Construal	Response Normalized Neg	0.021	0.07	0.012	0.295	–0.009	0.589
	Response Normalized Neu	–0.045	0.001	–0.014	0.318	0.031	0.111
	Response Normalized Pos	0.031	0.016	0.033	0.01	0.002	0.906
	Response Time Neg	–0.942	< 0.001^*^	–1.142	< 0.001^*^	–0.201	0.108
	Response Time Neu	–1.006	< 0.001^*^	–1.089	< 0.001^*^	–0.083	0.505
	Response Time Pos	–1.422	< 0.001^*^	–1.664	< 0.001^*^	–0.242	0.16
Memory Task	Neg c	–0.072	0.129	–0.092	0.054	–0.02	0.766
	Neg d-prime	–0.134	0.067	–0.061	0.401	0.073	0.482
	Neu c	–0.059	0.217	–0.071	0.137	–0.012	0.855
	Neu d-prime	0.015	0.847	0.042	0.597	0.027	0.811
	Pos c	–0.061	0.192	–0.078	0.099	–0.017	0.802
	Pos d-prime	–0.361	< 0.001^*^	–0.256	0.002^*^	0.104	0.359
Word Find	Find Time Neg	–0.024	0.201	–0.016	0.385	0.008	0.773
	Find Time Neu	–0.077	< 0.001^*^	–0.066	0.001^*^	0.011	0.7
	Find Time Pos	0.021	0.356	–0.004	0.855	–0.025	0.435
	Order Neg	–0.073	0.624	0.059	0.69	0.132	0.53
	Order Neu	–0.415	0.006	–0.308	0.04	0.107	0.613
	Order Pos	0.487	0.001	0.249	0.096	–0.239	0.256

The regression model was run twice with difference dummy code for intervention type. Change Across Negative Intervention represents the intercept when the positive intervention is coded as Pos = 1 and represents mean change in each DV across the negative intervention (when variables are standardized). Change Across Positive Intervention is the intercept when Pos = 0, and the Difference Between Intervention Conditions is the standardized regression coefficient that represents the effect of intervention condition (i.e., the difference between negative and positive conditions). ^*^ Represents significance after Bonferroni correction. DV = dependent variable. Neu = Neutral. Pos = Positive. Neg = Negative. C and d-prime are measures from signal detection theory (see Section “Materials and Methods”).

#### Physiology Variables

**FIGURE 3:**
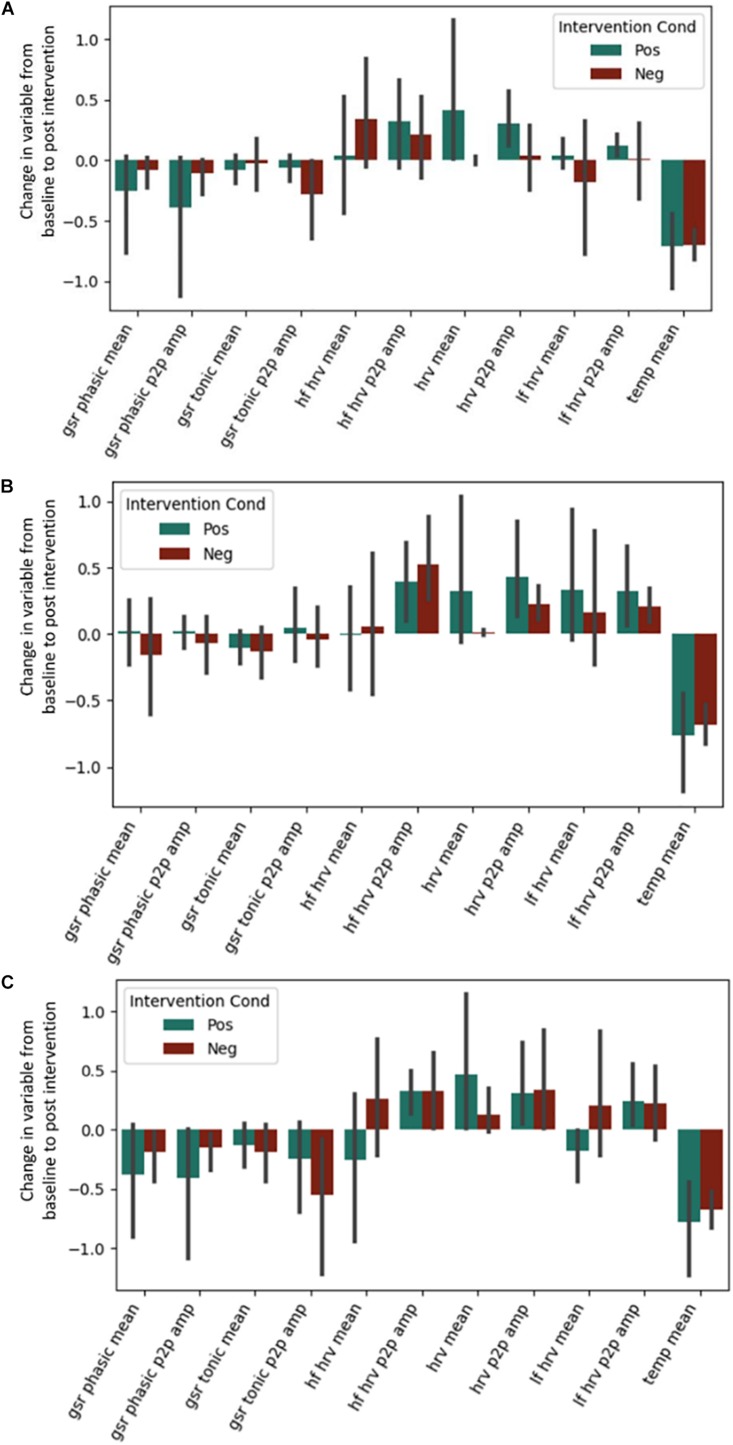
Post-intervention changes in autonomic variables during the **(A)** Questionnaire Task, **(B)** Emotional Memory Task, **(C)** Situation Construal task across the intervention by intervention type. GSR = Galvanic Skin Conductance. LF = Low Frequency. HF = High Frequency. HRV = Heart Rate Variability. Temp = Temperature. P2P = Peak to peak. No significant differences were found across intervention condition.

No effects of the intervention were observed for any of the autonomic physiological measures (see Figure 3). This includes the predicted High Frequency HRV, which was expected to be greater in the positive condition. Exact coefficients and *p*-values are available in Supplementary Material (see Supplementary Tables S3–S5).

#### EEG Variables

**FIGURE 4:**
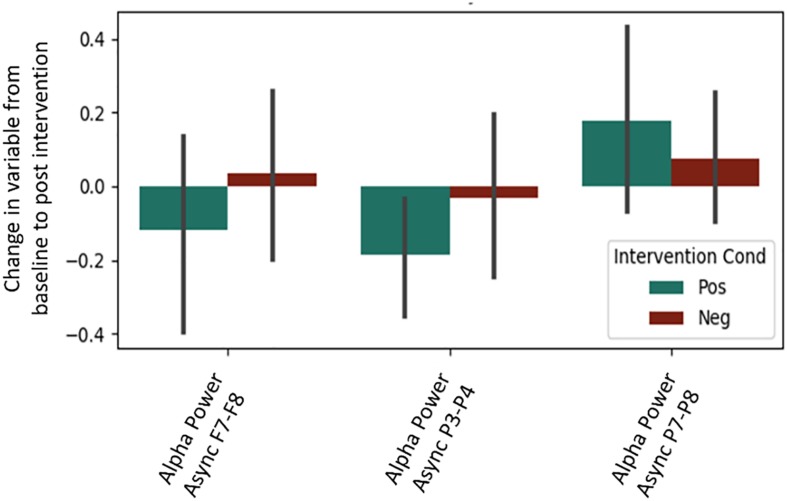
Post-intervention changes in EEG asynchrony variables by intervention type. No significant differences were found across intervention condition.

**FIGURE 5:**
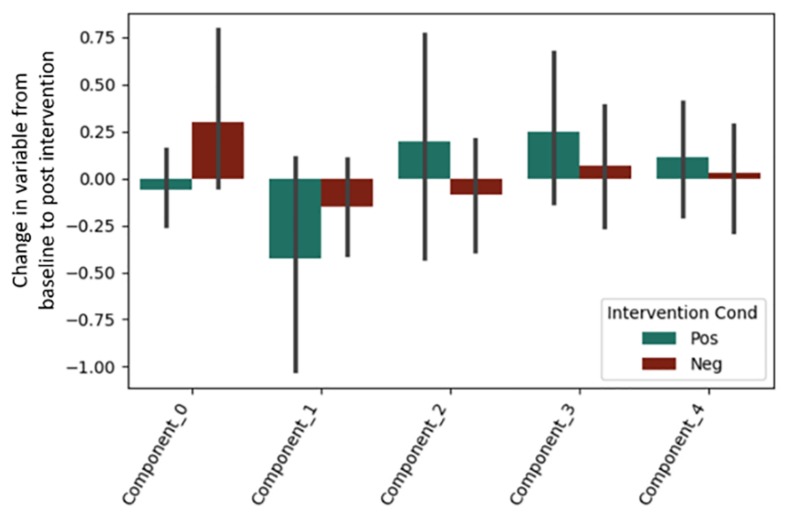
Post-intervention changes in EEG PCA components by intervention type. No significant changes were found across intervention condition.

It is important to note that a significant quantity of EEG data were lost due to experimenter error, poor signal connection, and/or artifact contamination. Only 35 participants (out of 86 total) are included in the EEG analysis; hence, the results should be treated as exploratory. We began by testing EEG frontal alpha asynchrony, a measure which has been used to quantify affect in the past (Coan and Allen, 2004; Wheeler et al., 2007). Prediction of the pre-post intervention difference from intervention type yielded no significant effects for asynchrony (Table 5 and Figure 4), although the direction of the effect matched that of previous literature, and the lack of significance may be due to low power. Analysis of 5 PCA components extracted from all EEG channels and measures also showed no differences between intervention conditions (Table 6 and Figure 5).

**TABLE 5:** Experiment 2 regression coefficients for EEG asynchrony.

	**Intercept**		**Intervention**	
		
**EEG Asynchrony DV (Positive Intervention = 1)**	**Coef**	***p***	**coef**	***p***
Alpha Power Async F7-F8	−1.29E-07	0.48	2.51E-07	0.28
Alpha Power Async P3-P4	−2.22E-07	0.29	3.49E-08	0.90
Alpha Power Async P7-P8	2.29E-07	0.16	−9.78E-08	0.63

**TABLE 6:** Experiment 2 regression coefficients for 5 EEG components.

	**Intercept**		**Intervention**	
		
**EEG Component DV (Positive Intervention = 1)**	**Coef**	***p***	**Coef**	***p***
Component 0	−0.001	0.779	0.006	0.188
Component 1	−0.043	0.053	0.028	0.325
Component 2	0.020	0.434	−0.029	0.392
Component 3	0.026	0.240	−0.019	0.505
Component 4	0.010	0.524	−0.007	0.722

### Machine Learning

#### Self-Report Variables

We then turned to a machine learning analysis, in which we predict the intervention condition from the change in measures between baseline and post-test. Our methods followed a hierarchical approach, in which we started with only self-report variables, and then added in cognitive, autonomic and EEG variables in a stepwise manner. The prediction accuracy using self-report variables acted as a baseline, where any increase above self-report accuracy with the addition of new variables meant that those new variables helped predict intervention condition, and were hence differentially affected by intervention type. Using self-report variables alone (with Studies 1 and 2 combined; see Table 7), we found logistic regression to be the best classifier, with an ability to predict the intervention condition from self-report variables at 74% (test accuracy).

**TABLE 7:** Train and test accuracy for machine learning models of self-report variables for Studies 1 and 2 combined.

**Model**	**Train**	**Train Std Error**	**Test**	**Test Std Err**
Logistic Regression	73%	1%	74%	<0.5%
SVM	76%	<0.5%	67%	<0.5%
Adaboost SVM	74%	<0.5%	68%	1%
Random Forests	80%	<0.5%	66%	1%

*SVM = Support Vector Machines. Std = Standard.*

The L1 regularization of logistic regression forces the beta weights of weakly predictive or highly co-linear measure to zero, thereby performing feature selection. Unsurprisingly, we found that the measures that were predictive in the univariate analysis were also predictive here: negative social emotions (β = –0.62), positive affect (β = 0.46), gratitude (β = 0.23) and elevation (β = 0.23). Optimism, which did not survive family-wise error correction in the original regression analysis, was also found to be predictive (β = 0.16). Other weakly predictive measures were competence, self-esteem: social, and meaning in life: search (β = 0.01, 0.01, and 0.02, respectively). Due to its high collinearity with positive affect and negative social emotions, negative affect, which was significant in the regression analysis, was not predictive here (β = 0).

We also ran the above analysis for Experiment 2 only, to aid later comparison with the physiological measures (see Table 8). Interestingly, classifiers trained on Experiment 2 had generally worse accuracy than both combined (best at 64% test accuracy). This was not due to the reduction in sample size, as the equivalent samples size (86 subjects, sampled randomly) produced equivalent accuracy to both datasets combined. The cause of this discrepancy is unknown, and includes the possibility that the discomfort, unfamiliarity, length, and solitude characterizing the EEG procedure and electrode cap led to dissipation of the psychological effects from the positive and negative interventions.

**TABLE 8:** Train and test accuracy for machine learning models of self-report variables (Experiment 2 only).

**Model**	**Train**	**Train Std Error**	**Test**	**Test Std Err**
Logistic Regression	66%	<0.5%	64%	<0.5%
SVM	68%	1%	52%	<0.5%
Adaboost SVM	68%	<0.5%	56%	<0.5%
Random Forests	86%	1%	57%	<0.5%

*SVM = Support Vector Machines. Std = Standard.*

#### Adding Cognitive Variables

Next, we added cognitive variables to determine if they aided in the detection of an intervention effect. However, because adding model predictor variables led to overfitting, we reduced the original set of 15 self-report variables to 5 using PCA before adding the cognitive variables. Using those five self-report PCA components alone, test accuracy was reduced by only 1% (to 73%). The addition of the 18 cognitive variables did not produce an increase in classification accuracy (instead, hurting performance; see Table 9).

**TABLE 9:** Train and test accuracy for machine learning models of cognitive variables.

**Model**	**Train**	**Train Std Error**	**Test**	**Test Std Err**
Logistic Regression	75%	<0.5%	69%	<0.5%
SVM	77%	1%	63%	<0.5%
Adaboost SVM	72%	<0.5%	64%	<0.5%
Random Forests	77%	1%	60%	<0.5%

*SVM = Support Vector Machines. Std = Standard.*

Given the reduction in performance, overfitting was likely, and therefore we reduced the number of predictors by also performing PCA on the cognitive components, and then added these to the self-report PCA components. The best test accuracy was again observed for logistic regression, but matched that of the self-report PCA components alone (72 ± 1%). Prediction of intervention condition from cognitive variables alone resulted in chance performance at test for all classifiers. We conclude that, similar to the regression analysis, machine learning analysis did not find evidence that cognitive variables were impacted by the positive or negative activity manipulation.

#### Adding Autonomic Variables

Physiological measures were collected during Experiment 2 only. To test if autonomic physiology variables added additional predictive power compared to self-report, we included 5 PCA components extracted from the autonomic variables to the PCA components from self-report, and then predicted intervention condition. Physiology was extracted from three tasks (questionnaire, emotional memory, situation construal), and each was added to the self-report variables in separate models (see Table 10). No models that included physiology and self-report (best test accuracy: 64 ± 4%) were better than self-report alone (test accuracy 64 ± 0.5%), and most resulted in chance performance. Further, models trained only on physiology resulted in chance test performance.

**TABLE 10:** Train and test accuracy for machine learning models of autonomic variables during the questionnaire task.

**Task**	**Model**	**Train**	**Train Std Error**	**Test**	**Test Std Err**
Questionnaire	Logistic Regression	75%	<0.5%	52%	<0.5%
	SVM	71%	1%	60%	2%
	Adaboost SVM	65%	<0.5%	60%	1%
	Random Forests	86%	1%	59%	1%
Emotional Memory	Logistic Regression	60%	1%	50%	1%
	SVM	65%	<0.5%	53%	2%
	Adaboost SVM	N/A	N/A	N/A	N/A
	Random Forests	78%	<0.5%	50%	2%
Situation Construal	Logistic Regression	65%	<0.5%	52%	<0.5%
	SVM	61%	<0.5%	50%	1%
	Adaboost SVM	N/A	N/A	N/A	N/A
	Random Forests	74%	1%	65%	4%

*SVM = Support Vector Machines. Std = Standard.*

#### EEG

Prediction of the intervention condition from EEG components yielded chance performance for all models. This is not surprising given the low sample size.

#### Relation Between Cognitive, Physiological and Self Report Variables

To investigate if cognitive and physiological measures are correlated with self-report variables in the context of our intervention, we ran series of models predicting each self-report measure from cognitive or physiological measures. We found one significant model, where Positive Affect was predicted by physiological variables during the emotional memory task (Table 11). Significant predictors of Positive Affect were Tonic GSR (Peak to Peak Amplitude) and the mean of High Frequency HRV.

**TABLE 11:** Regression coefficients for predicting positive affect from physiological variables during emotional memory task.

**IV**	**Coef**	***p***
Intercept	–0.132	0.005^*^
GSR phasic mean	63.245	0.012
GSR phasic P2P amp	–37.106	0.012
GSR tonic mean	0.153	0.463
GSR tonic P2P amp	–47.391	0.002^*^
HF HRV mean	0.011	0.002^*^
HF HRV P2P amp	0.000	0.542
LF HRV mean	0.000	0.960
LF HRV P2P amp	0.000	0.154
Temp mean	–0.036	0.055

Total model *F* = 3.4, *p* = 0.002, Variance explained = 37%. HRVL Heart Rate Variability, HF: High Frequency, LF: Low Frequency, P2P: Peak to Peak, GSR: Galvanic Skin Conductance, IV: Independent Variable. ^*^Represents a significant difference after Bonferroni correction.

## Conclusion and Discussion

Well-being science, and the field of social psychology in general, have traditionally focused on self-report to measure target variables (Baumeister et al., 2007). However, the potential bias apparent in these measures, even when rigorously validated, has propelled interest in alternative, more objective approaches. Here we investigated the efficacy of cognitive and physiological measures in determining the effect of positive versus negative activity interventions on well-being. While self-report variables produced significant results, we were unable to detect robust effects of well-being change in any cognitive or physiological measures. Our null results were possibly the results of chance, low power, or methodological limitations (see below), and we cannot conclusively establish whether our intervention had no cognitive or physiological effect. Additionally, we found limited evidence for a relation between any individual cognitive or physiological measures and any specific self-report variable. If power issues limited our physiological or cognitive machine learning findings, then the lack of these self-report and physio/cognitive relationships suggests that self-report measures an orthogonal component of well-being compared to these alternate measures. In conclusion, we suggest that these relatively more “objective” measures are not the silver bullet, set to revolutionize social psychological and well-being science, as purported by some (Pietro et al., 2014).

Although we did not detect significant effects of positive versus negative activity manipulations using cognitive and psychological measures, it is important to note some of the limitations of our approach and outline recommendations for future work.

### Physiological Data Collection Is Noisy and Restrictive and May Not Map to Affective Valence

While considered more objective, biological indicators are not themselves unambiguous measures of human happiness or unhappiness (Oswald and Wu, 2010). Previous theoretical work has argued that only arousal levels, and not the valence of emotion, are detectable via physiology (Schachter and Singer, 1962). In our design, we pitted induced negative affect versus positive affect, and, while the valences were clearly different, the arousal levels may have been similar, resulting in a null effect. We quantified physiological measures while subjects performed cognitive tasks, thereby allowing for event driven analysis. Although we strived for low motion artifacts, there may have been “artifacts” produced by task relevant cognition that masked our ability to detect affect change. We therefore recommend future studies also record physiological measures during a prolonged period of low arousal, where no stimulus is present. Further, physiological signals are inherently noisy, and prone to interference from movement, sweat, or electromagnetic sources. Future investigators should be cognizant of their low signal to noise ratio, and the need for substantial preprocessing (i.e., filtering, artifact rejection, dimensionality reduction, feature extraction, etc. Each preprocessing step demands careful consideration of a number of method “tuning” parameters, which reduces the accessibility of these methods to the average social scientist. Furthermore, reducing noise sources (e.g., via electrostatically shielded rooms or limited participant movement) may place undue restrictions on lab-based PAI experimentation, and certainly limit field studies.

### Research-Grade Devices Should Be Preferred

The devices used in our research (Feel wristband, Modified Emotive Epoch), while low-cost and wireless, were found to be limiting for rigorous research methodology. Neither device had the ability to embed stimulus triggers. The Feel device was synced with the stimulus computer, and it was assumed that its internal clock was robust and remained synchronized throughout the experimentation procedure. Furthermore, piloting revealed that the Emotiv device was becoming unsynchronized during the experimentation procedure. This was overcome by co-opting two EEG channels as a bipolar stimulus channel, driven by a photodiode connected to the stimulus presentation computer. It is possible that the addition of the synchronizing photodiode added artifacts that were not removed by our artifact rejection procedure, and this may have influenced our results. Further, the low sample rate (128 Hz) of the device may have hidden effects. The combination of numerous operational errors experienced with the device and excessive noise resulted in more than half of participants’ data being lost. Hence, the lack of significant EEG results may be due to low sample size, poor device quality, or artifacts. While considerable care was taken to ensure all signals were synchronized and denoised, the process was time consuming, and much could have been mitigated with the use of dedicated research grade devices.

### Limitations of Measures

Although we selected self-report, cognitive and physiological measures based on their prior utility and theory, our goal was not to develop a model that unifies self-report and physiological measures. However, we note the lack of any published theoretical model linking these measures. We also recognize that because the manipulation changes many correlated constructs, it is hard to make conclusions about the specific relationship between any one self-report construct and any specific physiological/cognitive measure. A useful addition to the field would be a set of repeated measures studies to determine the relationship between these measures in a variety of contexts, focusing on measure reliability, as well as their convergent and discriminate validity.

### Appropriate Control of Overfitting Should Be Employed

Due to the exploratory nature of this study, a model-free analysis approach was taken, where variables were combined using a number of machine learning techniques to predict the intervention (positive vs. negative) condition. We made heavy use of cross validation techniques to alleviate the possibility of overfitting (or false positives). We highlight this as especially important, because due to the sheer number of variables produced by each device, and the number of statistical comparisons that could be made, false positives are highly probable. Previous literature highlights the inconsistency of physiology in the detection of affect, as well as the possibility of Type 1 errors (given that, in each Experiment, a different set of variables was found to be predictive; Calvo and Mello, 2015). Therefore, we recommend future studies use appropriate methods to protect against spurious results coupled with appropriate power analysis. Another promising avenue includes model-based analyses, such as those employed in Bayesian cognitive modeling (M. D. Lee and Wagenmakers, 2014). Here, a number of specific theories that build in biologically plausible relations as priors (as parameterized by models) can be compared, and conclusions drawn are graded, where one theory is favored X time more than the other, as opposed to the all or nothing null hypothesis testing approach.

### Effect Is Small and Highly Variable

We found evidence of small effect sizes in our research, such that machine learning conducted on psychological variables could only predict the intervention condition with 64% accuracy (Revord et al., 2019). If we take the self-report variables as unbiased, and a small effect as given, then this small effect may simply not have been sufficient to produce a detectable cognitive and physiological response. Future studies should increase power via a larger sample size, or attempt to increase intervention effect either by including additional well-being activities [e.g., counting ones blessings (Emmons and McCullough, 2003), practicing optimism (King, 2001), performing acts of kindness (Nelson et al., 2016) random acts of kindness, using ones strengths in a new way (Seligman et al., 2005), affirming ones most import ant values (Nelson and Lyubomirsky, 2014) or meditating on positive feeling toward oneself (Fredrickson et al., 2008)] or by expanding the design longitudinally and thereby detecting the cumulative effects of a weaker intervention. The low effect size also suggests high variance in our measures. Previous investigators have noted that physiological responses are trait like (O’Gorman and Horneman, 1979; Pinna et al., 2007), and may be dampened or exaggerated in some people (Gerritsen et al., 2003); and this may have contributed to increased variance and lack of discernable effect. We recommend the addition of a baseline task in future studies, where the physiological response to a large number of highly emotionally arousing stimuli is recorded in the same participants and used to verify a detectable physiological change. This approach would also allow for the exclusion of subjects with low physiological responses, as well as for individual tuning of methods (with the caveat that the results would not generalize to the full population). We include a table of means and standard deviations of all measures in Supplementary Material (Supplementary Tables S6–S11), as a means for future investigators to estimate the expected effect size and hence required sample size of future studies. Further, no neutral condition was included, in which, for example, participants might be asked to recall memories without any strong emotional component. As a result, we could only compare positive and negative conditions. If some measures respond only to the arousal component of emotion (as argued previously), then we would be unable to detect a difference. In conclusion, our study replicated previous research on the beneficial effects of writing about prosocial events as quantified by self-report. Furthermore, although we did not clearly demonstrate the cognitive effects of a PAI, neither did we obtain conclusive evidence for a lack of cognitive of physiological effects. Our study introduced a new line of inquiry about the robustness and durability of the effects of positive interventions on the brain and body, which could lead to insights in determining the optimal “dosage” of kindness and gratitude interventions. We believe this paper represents a first step in introducing more cognitive paradigms into the positive activity literature, and set a precedent for the use of more objective measures in such research. However, in light of limitations of the methods and study design, we recommend that the field considers these measures, while bearing in mind our recommendations for future work. More research is needed to investigate the conditions under which these measures may be feasible and useful, but we stress that they should not be unilaterally favored over the traditional self-report approach.

## Data Availability

The datasets generated for this study are available on request to the corresponding author.

## Ethics Statement

Students reviewed and signed a consent form which detailed the study procedures. This consent form was reviewed and approved by the University of California, Riverside Institutional Review Board.

## Author Contributions

BY contributed to conceptualization, methodology, data curation, formal analysis, investigation, literature review, visualization, and manuscript preparation. JR contributed to conceptualization, methods, methodology, investigation, and literature review. SM contributed to conceptualization, methodology, and investigation. SL and AS initially conceived the study, administrated the study, and oversaw methods and investigation. All authors edited and approved the final manuscript.

## Conflict of Interest Statement

The authors declare that the research was conducted in the absence of any commercial or financial relationships that could be construed as a potential conflict of interest.
