# Supplementary material for: Cognitive and Physiological Measures in Well-Being Science: Limitations and Lessons
Source: Front Psychol. 2019 Jul 12;10:1630. doi: 10.3389/fpsyg.2019.01630 (PMC6640165; doi:10.3389/fpsyg.2019.01630)
Supplement: Supplementary file 1 [file Data_Sheet_1.docx]

Supplementary Material

# Supplementary Text

**S1 Text**

**Positive writing condition*.*** In the positive writing condition, participants were given a single piece of lined paper and instructed to free respond to two positive prosocial prompts (displayed on screen). The first prosocial prompt (gratitude writing) asked students to write about a kindness someone else had done to them. The prompt read as follows:

*Please take a moment to think back over the past several years of your life and remember an instance when someone did a kind, considerate, or thoughtful act (or acts) for you for which you are still grateful. Think of the people—parents, relatives, friends, neighbors, teachers, employers, and so on—who have been especially generous and thoughtful towards you. For example, you may feel grateful for a friend who was there when you needed them, or helped give you a new perspective on things when you were upset. Now, for the next 5 minutes, write about the kind, considerate, or thoughtful act (or acts) someone did for you for which you are still grateful.*

In the second prosocial prompt, participants were instructed to write about kind, considerate, or thoughtful acts they had done for someone else (i.e., a reflecting on one’s own kindness prompt), and how it affected that person’s life. The instructions were a modified version of the first prompt, with the following exceptions:

*Remember an instance when you did a kind, considerate, or thoughtful act (or acts) towards someone and how it made them feel… For example, you may have been there for a friend when they needed you, or helped give them a new perspective on things when they needed your support.*

**Negative writing condition*.*** The negative writing condition presented the parallel two writing prompts as the positive condition, except with negative or antisocial acts. The first negative (antisocial) prompt required participants to write about an unkindness someone else had done to them (i.e., recalling others’ unkind acts towards self). This prompt read as follows:

*Remember an instance when someone did a selfish, inconsiderate, or thoughtless act (or acts) towards you about which you are still upset…. For example, a friend may have been disloyal to you for his/her own benefit, or reluctant to help you when you needed them.*

In the second negative (antisocial) prompt, participants were instructed to write about an unkind act they had done to someone else (i.e., a reflecting on one’s own unkindness prompt), and how it affected that person’s life. The instructions were as follows:

*Remember an instance when you did a selfish, inconsiderate, or thoughtless act (or acts) towards someone and how it made them feel… For example, you may have been disloyal to a friend for your own benefit, or reluctant to help them when they needed you.*

# Supplementary Figures and Tables

## Supplementary Tables

*Table S1: Study 1 regression coefficients and p-values for each univariate regression.*

| Study 1  DV (Positive Intervetion=1) | Intercept | | Intervention | |
| --- | --- | --- | --- | --- |
|  | coeff | p | coef | p |
| Response Normalized Neg | 0.04 | 0.01 | -0.03 | 0.23 |
| Response Normalized Neu | -0.06 | 0.00 | 0.02 | 0.39 |
| Response Normalized Pos | 0.04 | 0.01 | -0.01 | 0.61 |
| Response Time Neg | -0.81 | 0.00 | -0.43 | 0.01 |
| Response Time Neu | -0.78 | 0.00 | -0.15 | 0.30 |
| Response Time Pos | -1.42 | 0.00 | -0.20 | 0.30 |
| Neg c | -0.08 | 0.12 | -0.05 | 0.46 |
| Neg dprime | -0.08 | 0.34 | 0.13 | 0.26 |
| Neu c | -0.07 | 0.16 | 0.00 | 0.97 |
| Neu dprime | -0.03 | 0.70 | 0.06 | 0.58 |
| Pos c | -0.06 | 0.22 | -0.03 | 0.70 |
| Pos dprime | -0.40 | 0.00 | 0.14 | 0.26 |
| Find Time Neg | -0.02 | 0.50 | 0.00 | 0.93 |
| Find Time Neu | -0.08 | 0.00 | 0.02 | 0.55 |
| Find Time Pos | 0.03 | 0.26 | -0.03 | 0.43 |
| Order Neg | -0.01 | 0.96 | -0.02 | 0.94 |
| Order Neu | -0.50 | 0.01 | 0.25 | 0.36 |
| Order Pos | 0.51 | 0.01 | -0.23 | 0.40 |

*Note: For study 1, we found no effect if the intervention type on any of the measures except the situation construal response time for negative items (coeff=-0.43, p<0.01), where in the positive intervention condition, subjects were significantly slower to find negative words. This result did not survive family wise error rate correction (Bonferroni method, correcting for 18 variables).*

*Table S2: Study 2 regression coefficients and p-values for each univariate regression.*

| Study 2 DV (Positive Intervetion=1) | Intercept | | Intervention | |
| --- | --- | --- | --- | --- |
|  | coeff | p | coef | p |
| Response Normalized Neg | -0.01 | 0.51 | 0.02 | 0.35 |
| Response Normalized Neu | -0.02 | 0.36 | 0.05 | 0.19 |
| Response Normalized Pos | 0.01 | 0.61 | 0.02 | 0.46 |
| Response Time Neg | -1.19 | 0.00 | 0.21 | 0.33 |
| Response Time Neu | -1.44 | 0.00 | 0.09 | 0.68 |
| Response Time Pos | -1.43 | 0.00 | -0.31 | 0.36 |
| Neg c | -0.06 | 0.55 | 0.03 | 0.82 |
| Neg dprime | -0.25 | 0.09 | -0.01 | 0.96 |
| Neu c | -0.04 | 0.68 | -0.03 | 0.83 |
| Neu dprime | 0.11 | 0.54 | -0.05 | 0.84 |
| Pos c | -0.06 | 0.55 | 0.00 | 0.98 |
| Pos dprime | -0.28 | 0.10 | 0.04 | 0.87 |
| Find Time Neg | -0.04 | 0.21 | 0.03 | 0.53 |
| Find Time Neu | -0.07 | 0.02 | -0.01 | 0.85 |
| Find Time Pos | 0.00 | 0.94 | -0.01 | 0.87 |
| Order Neg | -0.20 | 0.43 | 0.40 | 0.25 |
| Order Neu | -0.24 | 0.32 | -0.15 | 0.66 |
| Order Pos | 0.44 | 0.06 | -0.25 | 0.45 |

*Table S3: Univariates for physiological regressions - Questionnaires*

| Questionnaire: DV | Intercept (Pos=1) | | Intercept (Pos=0) | | Intervention | |
| --- | --- | --- | --- | --- | --- | --- |
|  | Coef | p | Coef | p | Coef | p |
| GSR phasic mean (uS) | -0.001 | 0.629 | -0.002 | 0.142 | -0.001 | 0.474 |
| GSR phasic p2p amp (uS) | -0.001 | 0.662 | -0.004 | 0.126 | -0.003 | 0.429 |
| GSR tonic mean (uS) | -0.006 | 0.779 | -0.008 | 0.741 | -0.001 | 0.969 |
| GSR tonic P2P amp (uS) | -0.001 | 0.075 | 0 | 0.654 | 0.002 | 0.117 |
| HF HRV mean (ms^2^) | 0.659 | 0.154 | 0.078 | 0.867 | -0.581 | 0.375 |
| HF HRV p2p amp (ms^2^) | 107.926 | 0.228 | 191.369 | 0.038 | 83.443 | 0.512 |
| LF HRV mean (ms^2^) | -4.904 | 0.405 | 1.088 | 0.858 | 5.992 | 0.479 |
| LF HRV P2P amp (ms^2^) | 19.023 | 0.905 | 160.975 | 0.331 | 141.952 | 0.537 |
| Temp mean (*C) | -1.525 | 0 | -1.545 | 0 | -0.02 | 0.959 |

*Table S4: Univariates for physiological regressions – Emotional Memory*

| Emotional Memory: DV | Intercept (Pos=1) | | Intercept (Pos=0) | | Intervention | |
| --- | --- | --- | --- | --- | --- | --- |
|  | Coef | p | Coef | p | Coef | p |
| GSR phasic mean (uS) | -0.001 | 0.566 | 0.001 | 0.263 | 0.002 | 0.234 |
| GSR phasic p2p amp (uS) | -0.001 | 0.593 | 0.002 | 0.227 | 0.002 | 0.22 |
| GSR tonic mean (uS) | -0.041 | 0.123 | -0.03 | 0.238 | 0.01 | 0.782 |
| GSR tonic P2P amp (uS) | 0 | 0.734 | 0 | 0.727 | 0.001 | 0.627 |
| HF HRV mean (ms^2^) | -0.553 | 0.665 | -0.032 | 0.979 | 0.522 | 0.767 |
| HF HRV p2p amp (ms^2^) | 258.082 | 0.001 | 191.172 | 0.013 | -66.909 | 0.534 |
| LF HRV mean (ms^2^) | 2.724 | 0.579 | 5.642 | 0.246 | 2.918 | 0.672 |
| LF HRV P2P amp (ms^2^) | 229.523 | 0.096 | 352.39 | 0.011 | 122.867 | 0.522 |
| Temp mean (*C) | -1.404 | 0 | -1.561 | 0 | -0.157 | 0.71 |

*Table S5: Univariates for physiological regressions – Situation Construal*

| Situation Construal: DV | Intercept (Pos=1) | | Intercept (Pos=0) | | Intervention | |
| --- | --- | --- | --- | --- | --- | --- |
|  | Coef | p | Coef | p | Coef | p |
| GSR phasic mean (uS) | -0.001 | 0.336 | -0.002 | 0.058 | -0.001 | 0.5 |
| GSR phasic p2p amp (uS) | -0.002 | 0.453 | -0.005 | 0.049 | -0.003 | 0.379 |
| GSR tonic mean (uS) | -0.044 | 0.27 | -0.07 | 0.073 | -0.027 | 0.627 |
| GSR tonic P2P amp (uS) | -0.003 | 0.057 | -0.003 | 0.061 | 0 | 0.966 |
| HF HRV mean (ms^2^) | 0.681 | 0.379 | -0.662 | 0.392 | -1.343 | 0.221 |
| HF HRV p2p amp (ms^2^) | 200.925 | 0.007 | 189.771 | 0.011 | -11.154 | 0.913 |
| LF HRV mean (ms^2^) | 8.668 | 0.346 | -7.535 | 0.42 | -16.203 | 0.218 |
| LF HRV P2P amp (ms^2^) | 242.772 | 0.148 | 259.656 | 0.123 | 16.884 | 0.943 |
| Temp mean (*C) | -1.384 | 0 | -1.602 | 0 | -0.217 | 0.609 |

*Table S6: Means (sd) for cognitive measures*

| DV | Pre Neg Intervention | Post Neg Intervention | Pre Pos Intervention | Post Pos Intervention |
| --- | --- | --- | --- | --- |
| Find Time Neg (s) | 0.639 (0.143) | 0.615 (0.146) | 0.646 (0.143) | 0.628 (0.155) |
| Find Time Neu (s) | 0.613 (0.173) | 0.535 (0.145) | 0.619 (0.154) | 0.555 (0.140) |
| Find Time Pos (s) | 0.507 (0.181) | 0.529 (0.175) | 0.475 (0.167) | 0.471 (0.159) |
| Neg c | 0.404 (0.585) | 0.337 (0.617) | 0.353 (0.688) | 0.272 (0.692) |
| Neg dprime | 1.207 (0.740) | 1.074 (0.730) | 1.155 (0.738) | 1.088 (0.774) |
| Neu c | 0.751 (0.517) | 0.695 (0.572) | 0.687 (0.594) | 0.622 (0.647) |
| Neu dprime | 1.201 (0.840) | 1.212 (0.727) | 1.249 (0.737) | 1.286 (0.885) |
| Pos c | 0.502 (0.653) | 0.448 (0.672) | 0.382 (0.704) | 0.307 (0.738) |
| Pos dprime | 1.107 (0.781) | 0.744 (0.710) | 1.001 (0.783) | 0.744 (0.648) |
| Order Neg | 4.521 (1.172) | 4.446 (1.176) | 4.619 (1.156) | 4.661 (1.177) |
| Order Neu | 4.196 (1.299) | 3.774 (1.108) | 4.277 (1.122) | 3.983 (0.999) |
| Order Pos | 3.283 (1.220) | 3.780 (1.248) | 3.105 (1.036) | 3.356 (1.117) |
| Response Normalized Neg | -0.288 (0.113) | -0.268 (0.114) | -0.265 (0.131) | -0.252 (0.117) |
| Response Normalized Neu | -0.004 (0.099) | -0.048 (0.119) | -0.005 (0.109) | -0.021 (0.117) |
| Response Normalized Pos | 0.143 (0.116) | 0.174 (0.098) | 0.149 (0.098) | 0.182 (0.109) |
| Response Time Neg (s) | 3.691 (1.024) | 2.751 (0.775) | 3.919 (1.320) | 2.769 (0.875) |
| Response Time Neu (s) | 3.774 (1.165) | 2.759 (0.843) | 3.869 (1.298) | 2.768 (0.867) |
| Response Time Pos (s) | 4.053 (1.348) | 2.633 (0.819) | 4.271 (1.660) | 2.592 (0.746) |

*Table S7: Normalized means (sd) for self-report measures*

| DV | Pre Neg Intervention | Post Neg Intervention | Pre Pos Intervention | Post Pos Intervention |
| --- | --- | --- | --- | --- |
| Autonomy | 0.193 (0.283) | 0.176 (0.269) | 0.192 (0.319) | 0.208 (0.322) |
| Competence | 0.146 (0.337) | 0.113 (0.323) | 0.134 (0.352) | 0.152 (0.344) |
| Connectedness | 0.267 (0.326) | 0.243 (0.341) | 0.315 (0.348) | 0.338 (0.373) |
| Elevation | -0.098 (0.370) | -0.121 (0.374) | -0.081 (0.385) | 0.086 (0.425) |
| Gratitude | 0.268 (0.481) | 0.103 (0.583) | 0.342 (0.502) | 0.385 (0.488) |
| Meaning in Life: Presence | 0.223 (0.365) | 0.199 (0.383) | 0.292 (0.349) | 0.295 (0.353) |
| Meaning in Life: Search | 0.386 (0.404) | 0.364 (0.461) | 0.316 (0.475) | 0.312 (0.487) |
| Negative Affect | -0.491 (0.369) | -0.422 (0.358) | -0.483 (0.370) | -0.570 (0.361) |
| Negative Social Emotions | -0.729 (0.264) | -0.488 (0.469) | -0.682 (0.346) | -0.715 (0.338) |
| Optimism | 0.091 (0.227) | 0.068 (0.218) | 0.093 (0.221) | 0.112 (0.222) |
| Positive Affect | -0.075 (0.372) | -0.253 (0.406) | -0.065 (0.382) | 0.015 (0.413) |
| Self Esteem: Appearance | 0.069 (0.395) | 0.079 (0.387) | 0.146 (0.388) | 0.205 (0.369) |
| Self Esteem: Performance | 0.245 (0.338) | 0.220 (0.343) | 0.213 (0.351) | 0.256 (0.343) |
| Self Esteem: Social | 0.122 (0.416) | 0.122 (0.437) | 0.176 (0.388) | 0.239 (0.393) |

*Table S8: Means (sd) for physiological measures during emotional memory*

| DV | Pre Neg Intervention | Post Neg Intervention | Pre Pos Intervention | Post Pos Intervention |
| --- | --- | --- | --- | --- |
| GSR phasic mean (uS) | 0.004 (0.004) | 0.004 (0.004) | 0.003 (0.003) | 0.004 (0.005) |
| GSR phasic p2p amp (uS) | 0.003 (0.005) | 0.002 (0.004) | 0.005 (0.017) | 0.003 (0.005) |
| GSR tonic mean (uS) | 0.247 (0.255) | 0.222 (0.193) | 0.238 (0.325) | 0.254 (0.403) |
| GSR tonic P2P amp (uS) | 0.002 (0.002) | 0.001 (0.003) | 0.003 (0.006) | 0.003 (0.009) |
| HF HRV mean (ms^2^) | 1.362 (4.222) | 1.399 (8.401) | 0.672 (5.454) | 0.641 (5.120) |
| HF HRV p2p amp (ms^2^) | 649.666 (436.481) | 907.747 (667.417) | 508.702 (327.603) | 699.874 (388.831) |
| LF HRV mean (ms^2^) | -1.152 (11.307) | 1.572 (17.126) | -0.198 (4.124) | 5.444 (26.775) |
| LF HRV P2P amp (ms^2^) | 486.269 (833.755) | 715.792 (1107.911) | 350.057 (735.205) | 702.447 (1546.370) |
| Temp mean (*C) | 36.095 (1.167) | 34.691 (1.704) | 36.047 (1.690) | 34.486 (2.783) |

*Table S9: Means (sd) for physiological measures during situation construal*

| DV | Pre Neg Intervention | Post Neg Intervention | Pre Pos Intervention | Post Pos Intervention |
| --- | --- | --- | --- | --- |
| GSR phasic mean (uS) | 0.004 (0.004) | 0.003 (0.001) | 0.006 (0.011) | 0.004 (0.004) |
| GSR phasic p2p amp (uS) | 0.004 (0.006) | 0.002 (0.001) | 0.009 (0.024) | 0.004 (0.008) |
| GSR tonic mean (uS) | 0.242 (0.264) | 0.214 (0.192) | 0.229 (0.235) | 0.199 (0.248) |
| GSR tonic P2P amp (uS) | 0.005 (0.010) | 0.001 (0.001) | 0.003 (0.006) | 0.002 (0.003) |
| HF HRV mean (ms^2^) | -0.125 (2.042) | 0.555 (3.181) | 0.068 (2.274) | -0.630 (2.831) |
| HF HRV p2p amp (ms^2^) | 874.522 (592.022) | 1004.504 (634.323) | 583.864 (232.586) | 748.694 (300.504) |
| LF HRV mean (ms^2^) | 3.020 (17.835) | 11.689 (76.568) | 1.877 (9.529) | -7.249 (27.461) |
| LF HRV P2P amp (ms^2^) | 702.003 (1083.361) | 903.104 (1105.821) | 477.581 (1179.430) | 535.477 (1000.406) |
| Temp mean (*C) | 36.069 (1.179) | 34.685 (1.719) | 36.025 (1.708) | 34.349 (2.750) |

*Table S10: Means (sd) for physiological measures during questionnaire*

| DV | Pre Neg Intervention | Post Neg Intervention | Pre Pos Intervention | Post Pos Intervention |
| --- | --- | --- | --- | --- |
| GSR phasic mean (uS) | 0.004 (0.004) | 0.003 (0.002) | 0.006 (0.015) | 0.003 (0.001) |
| GSR phasic p2p amp (uS) | 0.003 (0.005) | 0.002 (0.003) | 0.005 (0.018) | 0.002 (0.001) |
| GSR tonic mean (uS) | 0.232 (0.242) | 0.226 (0.199) | 0.222 (0.309) | 0.174 (0.197) |
| GSR tonic P2P amp (uS) | 0.002 (0.005) | 0.001 (0.002) | 0.003 (0.008) | 0.001 (0.002) |
| HF HRV mean (ms^2^) | -0.635 (1.510) | 0.024 (2.101) | -0.131 (2.519) | -0.027 (1.453) |
| HF HRV p2p amp (ms^2^) | 807.356 (552.126) | 915.282 (648.331) | 570.396 (340.675) | 745.598 (360.100) |
| LF HRV mean (ms^2^) | 1.134 (40.752) | -3.770 (24.749) | 1.800 (12.721) | 0.570 (22.750) |
| LF HRV P2P amp (ms^2^) | 776.118 (1406.140) | 795.140 (1149.385) | 613.276 (1612.623) | 575.204 (1092.570) |
| Temp mean (*C) | 35.885 (1.339) | 34.360 (1.873) | 35.762 (1.800) | 34.152 (2.876) |

Table S11: Means (sd) for EEG Asynchrony Measures

| DV | Pre Neg Intervention | Post Neg Intervention | Pre Pos Intervention | Post Pos Intervention |
| --- | --- | --- | --- | --- |
| Alpha Power Async F7-F8 (power*1e6) | -0.861 (1.106) | -0.720 (1.358) | 0.000 (1.080) | -0.580 (1.496) |
| Alpha Power Async P3-P4  (power*1e6) | -0.482 (1.143) | -0.662 (1.542) | -0.536 (1.213) | -0.758 (1.289) |
| Alpha Power Async P7-P8  (power*1e6) | 0.089 (1.689) | 0.221 (1.798) | -0.820 (0.768) | -0.591 (0.865) |

## Supplementary Figures

| Note: All non-significant.  *Figure S1: Study 1 change in normalized self-report measures across intervention*  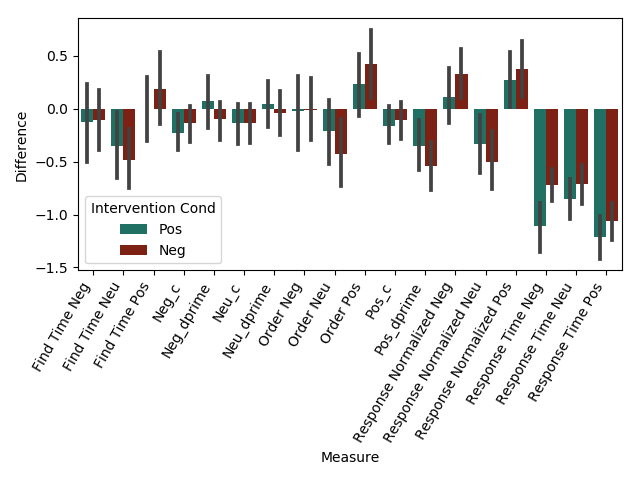  * |
| --- |
| *Figure S2: Study 2 change in normalized self-report measures across intervention* 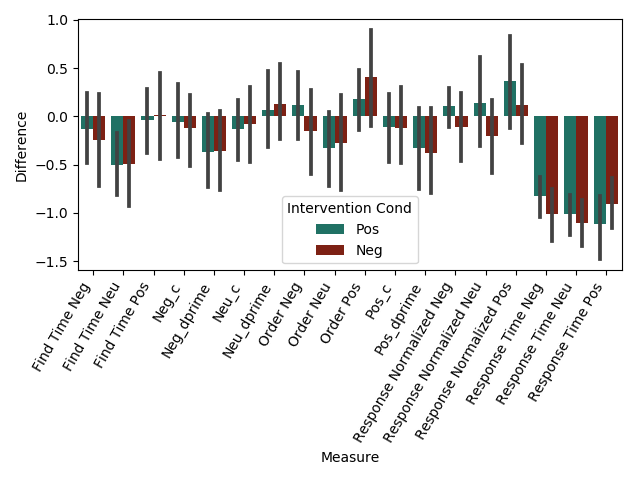  * |
